# Endogenous suspension of phenomenal consciousness during extended cessation: Regional homogeneity analysis of multi-participant 7T fMRI of an advanced meditative endpoint

**DOI:** 10.1162/IMAG.a.1443

**Published:** 2026-11-05

**Authors:** Winson F.Z. Yang, Akila Kadambi, Ruby Potash, Terje Sparby, Matthew D. Sacchet

**Affiliations:** Meditation Research Program, Department of Psychiatry, Massachusetts General Hospital, Harvard Medical School, Boston, MA, United States; Athinoula A. Martinos Center for Biomedical Imaging, Department of Radiology, Massachusetts General Hospital, Harvard Medical School, Boston, MA, United States; Department of Psychiatry and Biobehavioral Sciences, David Geffen School of Medicine, University of California, Los Angeles, CA, United States; Brain and Creativity Institute, Dornsife College of Letters, Arts, and Sciences, University of Southern California, Los Angeles, CA, United States; Steiner University College, Oslo, Norway; Department of Psychology and Psychotherapy, Witten/Herdecke University, Witten, Germany; Integrated Curriculum for Anthroposophic Psychology, Witten/Herdecke University, Witten, Germany

**Keywords:** advanced meditation, extended cessation, consciousness, subcortical, brainstem, chemoarchitecture

## Abstract

Extended cessation (EC) is a rare advanced meditative state characterized by the voluntary absence of reportable phenomenal experience, followed by reproducible aftereffects including immense mental clarity, equanimity, and peace. EC offers an endogenous model for investigating the mechanisms of consciousness. In a small-N case study (N = 3) using ultra-high-resolution 7T functional magnetic resonance imaging (fMRI) with dense within-subject sampling, we quantified whole-brain activity and related it to chemoarchitecture and cognitive maps. EC is marked by increased activity in unimodal sensory cortices and viscerosensory brainstem nuclei, and down-regulation in transmodal regions, subcortex and brainstem. Meta-analytic cognitive decoding linked EC to perceptual clarity and attentional processes, and away from distress-related domains. Multivariate coupling to normative neurotransmitter maps revealed that EC-related connectivity changes covaried with histaminergic H_3_ receptors topology. These findings constrain prevailing theories of consciousness by demonstrating that loss of experience need not entail global neural suppression, and supports accounts emphasizing selective precision modulation and hierarchical decoupling. More broadly, EC demonstrates that consciousness can cease without global suppression, suggesting a potential “reset” mechanism that fosters equanimity and the potential for flourishing.

## 1 Introduction

In most scientific and clinical contexts, wakefulness is treated as sufficient for ongoing subjective consciousness (Seth & Bayne, 2022). Even when perceptual content is minimal, conscious awareness is typically presumed to persist. However, reports from advanced meditation describe rare states in which perception is radically altered (Chowdhury et al., 2025; Yang et al., 2024), or even phenomenologically inaccessible for brief periods (Berkovich-Ohana, 2015; Chowdhury et al., 2023; van Lutterveld et al., 2024, 2025), or in rare cases, for extended periods (Laukkonen et al., 2023). These reports raise foundational questions about the relationship between wakefulness, brain activity, and conscious experience (Sacchet et al., 2024; Sparby & Sacchet, 2025).

A central challenge in the scientific study of consciousness concerns how the term “consciousness” is defined (Mudrik et al., 2025; Seth & Bayne, 2022). In this study, we operationalize consciousness as phenomenal consciousness, which is the presence of subjective experience, including perception, affective tone, and self-referential awareness reflecting implicit experiential ownership. Under this operational definition, consciousness may be temporarily absent even when the organism remains biologically alive and capable of later re-entering conscious experience. Importantly, this operationalization relies on retrospective reportability and reproducible post-state phenomenology, rather than direct online sampling during the state itself.

This distinction between biological wakefulness and phenomenal consciousness is made explicit in early Buddhist psychological frameworks. The meditative attainment traditionally referred to as *nirodha samāpatti* is defined as the cessation of feeling and perception, two mental factors regarded as universally present in conscious cognition (Gunaratana, 1985; Laukkonen et al., 2023; Nanamoli & Bodhi, 2009; Noriaki, n.d.). Within this phenomenological taxonomy, every moment of consciousness is understood to require both affective tone and perceptual recognition. Accordingly, nirodha samāpatti is described as a temporary suspension of all phenomenal experience, while physiological activity remains intact. Upon re-emergence, consciousness resumes and is reported to resume with marked clarity and equanimity. It is noteworthy that nirodha samāpatti is historically considered to be the highest meditative attainment within some Theravāda contemplative taxonomies (Griffiths, 1986; Lusthaus, 2002). Refer to Laukkonen et al. (2023) for a recent discussion of the historical and theoretical context of nirodha samāpatti.

Here, we study what we now call extended cessation (EC) in the scientific literature, without making definitive claims about its doctrinal interpretation. Historical accounts of nirodha samāpatti fall within this framework, but the present work is concerned with defining EC in operational, neuroscientifically testable terms rather than resolving its place within contemplative traditions. We define EC operationally as a voluntarily entered, temporally bounded state characterized by the absence of reportable phenomenal experience, followed by spontaneous re-emergence of consciousness and reproducible post-EC psychological aftereffects. Reported aftereffects include extreme clarity, equanimity, significant reduction in mental suffering, repetitive thoughts, and an absence of inner narration (Laukkonen et al., 2023). EC may vary from minutes to hours or longer and is entered and exited volitionally, distinguishing it from non-conscious states induced by pharmacological agents (e.g., anesthesia or psychedelics) (Pujol et al., 2021, 2022). Crucially, EC is defined here at the level of phenomenal consciousness only; it does not imply absence of brain activity, arousal, or neural processes supporting later recovery of experience.

From a neuroscience perspective, studying EC offers a rare opportunity to examine which neural mechanisms are necessary or insufficient for sustaining phenomenal consciousness (Pujol et al., 2021, 2022). A major limitation in current consciousness research is the absence of an endogenous and reversible method to suspend conscious experience while preserving physiological stability and enabling prospectively timed re-emergence (Gunaratana, 1985; Laukkonen et al., 2023). Most non-conscious states studied experimentally, such as sleep, anesthesia, and disorders of consciousness, are associated with global suppression of brain activity, reduced arousal, or pathological damage (Pujol et al., 2021, 2022). These exogenous methods complicate inferences about the neural basis of conscious experience itself. In contrast, EC is endogenous and precisely timed, enabling prospective examination of transitions into and out of non-experiential states. This operational definition does not presume that EC is categorically distinct from all other forms of non-experiential states; rather, it provides a constrained empirical model for studying transitions in phenomenal consciousness.

Several contemporary theories of consciousness emphasize distinct neural mechanisms thought to be necessary for sustaining phenomenal experience. Global Neuronal Workspace Theory (GNWT) highlights the role of long-range connectivity and feedback connectivity, particularly involving frontoparietal and thalamic systems, in enabling conscious access (Mashour et al., 2020). Integrated Information Theory (IIT) emphasizes neural differentiation and integration as prerequisites for consciousness (Tononi et al., 2016). In contrast, Predictive Processing and Active Inference (AIF) frameworks propose that conscious experience depends on the precision-weighted integration of sensory and conceptual signals, with thalamo-cortical gating mechanisms regulating whether representations enter conscious access (Laukkonen et al., 2025; Parr et al., 2022; Tal et al., 2026). Here, we do not provide tests of any single theoretical framework; rather, we provide an empirical context to examine which specific neural correlates emphasized by these accounts are present or attenuated during the suspension of phenomenal consciousness.

We note that EC, as operationally defined here, is inspired by the classical account of nirodha samāpatti. This framing is broader and is explicitly distinct from non-dual awareness, as described in traditions such as Mahamudra and Advaita Vedanta, in which perspectival ownership of experience is absent but phenomenal experience itself is not suspended (Dunne, 2011, 2015). EC and non-dual awareness, therefore, represent meaningfully distinct phenomenological categories, and the present study speaks only to EC. Furthermore, we make no claims about EC as a universal or singular meditative endpoint, nor about its primacy or sufficiency as a contemplative attainment. The present findings are constrained to this operational definition and do not speak to other contemplative endpoints.

Beyond its theoretical relevance, characterizing the neural correlates of EC may inform how transient suspension of phenomenal experience may reshape affective, motivational, and self-referential dynamics after the state resolves. Understanding these neural mechanisms provides a framework for explaining reported post-EC aftereffects. Such frameworks are essential for providing an empirical foundation for meditative endpoints toward translational models of resilience, adaptability, and human flourishing. These frameworks may also inform applications across domains concerned with mental flexibility and mental health, as well as for generating testable hypotheses in longitudinal research.

Here, we present the first neuroscientific, empirical investigation of EC, using ultra-high-resolution 7T functional magnetic resonance imaging (fMRI) to investigate the whole-brain activity during EC. The ultra-high spatial resolution of 7T imaging allows for precise measurement of small but critical brain structures that are central to consciousness and arousal regulation, such as the thalamus, brainstem, and subcortical nuclei. Moreover, we used a novel and systematic neurophenomenological approach to rigorously examine the subjective experience of EC, enabling a rare convergence of first-person insight and third-person measurement. Crucially, we intensively sampled each participant across repeated EC and control sessions, yielding rich within-individual time series for an appropriate small-N study design. To appropriately model these intensively sampled data, we used group-level statistical frameworks optimized for intensive within-individual designs, using linear mixed-effects models with explicit correction for temporal autocorrelation and repeated-measures dependence (Bolger & Laurenceau, 2013; Hamaker et al., 2005; McNeish & Hamaker, 2020). This statistical framework enables characterization of the neural correlates of EC and is particularly appropriate for a phenomenologically distinct and stereotyped state (Becker, 2023; Milne et al., 2018).

This small-N case study provides initial empirical evidence characterizing the neural correlates of EC. We sought to answer three key questions: (1) What are the neural correlates that accompany voluntary suspension of phenomenal consciousness?; (2) What cognitive architecture does EC most closely associate with?; and (3) Which neurochemical systems are implicated, as inferred from spatial coupling to normative receptor distributions? Together, these analyses aim to understand the neural correlates underlying phenomenal consciousness while acknowledging the methodological and epistemic limits inherent in studying rare, self-reported experiential states. More broadly, understanding the mechanisms of EC may help explain how meditative endpoints such as EC contribute to enduring changes in well-being, meaning, and human flourishing.

## 2 Materials and Methods

### 2.1 Participants

Participants were three male advanced meditators, Subject A (age 32 at time of data collection), Subject B (age 46 at time of data collection), and Subject C (age 66 at time of data collection). Important to the current study, the participants are advanced meditators and reported being able to incline toward EC as the target of meditation. Subject A had over 19 years of meditation experience with total meditation experience of an estimated at least 15,000 hours. Subject B had over 4 years of meditation experience, and an estimated total practice amount at least of 9,000 hours. Subject C had over 50 years of meditation experience with total meditation experience of at least 35,000 hours. Lifetime practice hours are approximate self-reports based on duration and frequency of weekly practice and retreats. We also note that lifetime hours are not necessarily a direct measure of meditative expertise. The Mass General Brigham IRB approved the study, and the participants provided informed consent.

### 2.2 Experimental design

#### 2.2.1 Extended cessation (EC)

FMRI data during EC was collected as part of a larger study of advanced meditation practices. In this advanced practice of EC, participants have trained extensively to develop the precision to enter and exit EC at specific, predefined times. This level of mastery reflects not only the ability to achieve a deep cessation state but also control over the duration and timing of the state. The participants performed EC meditation as the last meditation session after all other MRI sessions (structural scans, advanced concentrative absorption meditation, advanced insight meditation, non-meditative control conditions) were completed. Each participant completed three successive 15-minute EC fMRI sessions within a single scanner visit, beginning immediately after the preceding session concluded, yielding approximately 45 minutes of EC fMRI data per participant in total. During each session, participants were asked to meditate, enter, and maintain EC for the duration of the run.

Prior to each EC run, participants set an intention to remain in EC for approximately 15 minutes. This intention is formed before EC entry and does not constitute volitional monitoring during the state itself. The state, by definition, precludes ongoing phenomenal experience including intentional monitoring. This pre-entry intention-setting is, therefore, not in contradiction with the phenomenological definition of EC, but is analogous to setting a temporal anchor prior to entering a state in which such monitoring is unavailable. We acknowledge, however, that the mechanism by which this pre-entry intention regulates the duration of EC remains phenomenologically and neuroscientifically unresolved, and represents an important open question for future research.

Then the participant started meditating at the start of the EC run. All three participants provided a button press marking the moment of intended EC entry, that is, the point at which they began inclining toward EC. The run ended when the participant exited out of EC and indicated the exit with a button press. Retrospectively, we asked participants to estimate the duration it took them to enter EC if they did not indicate with a button press initially. Based on post-session phenomenological reports, participants described entering EC within seconds of the entry button press. To further mitigate potential bleeding effects at EC boundaries whereby pre-EC or post-EC neural dynamics might contaminate the EC epoch, as well as residual temporal uncertainty between the entry button press and the actual onset of phenomenal cessation, the first minute of fMRI volumes following the entry button press and the final minute of volumes preceding the exit button press were excluded from all analyses. The remaining data within each run were treated as the EC epoch for statistical analysis. We acknowledge that this procedure provides a close approximation of EC onset rather than a precise real-time marker of phenomenal cessation, as the exact moment of cessation cannot be reported until after the state has ended.

After completing the meditation task, all participants completed a standardized EC phenomenology questionnaire—designed to reflect how they typically practice and experience EC—covering preparation, onset, absence of consciousness, exit, and afterglow. Two participants provided their phenomenology responses after data collection had formally concluded, and their delayed reports have been included in the dataset.

#### 2.2.2 Non-meditative control conditions

We developed two non-meditative control conditions (hereon control conditions) that were used to compare against EC. These control conditions were designed to engage the participant’s mind with non-meditative mental activities. These conditions were carefully selected to be sufficiently engaging to not induce meditative states. We did not use a resting-state control condition, since experienced meditators may enter meditative states during this period (Tang et al., 2012).

The two different control conditions implemented in this study were: (1) a memory control condition in which participants were asked to reminisce the events of the past 1 week and narrate them sub-vocally in their minds, closing their eyes and without moving their lips, for 8 minutes; and a (2) counting control condition, where the participants were asked to mentally count down in decrements of 5 from 10,000 for 8 minutes, closing their eyes and without moving their lips. We collected two runs for each control condition, which provided 16 minutes data for each control condition. However, for Subject A, only a single run for each control condition was collected as part of another study.

### 2.3 Neuroimaging acquisition

Neuroimaging was acquired using a 7T MR scanner (SIEMENS MAGNETOM Terra) using a 32-channel head coil. Functional imaging was performed using a single-shot two-dimensional echo planar imaging sequence with T2*-weighted BOLD-sensitive MRI, repetition time (TR) = 2.9 seconds, echo time (TE) = 30 ms, flip angle (FA) = 75°, field of view (FOV) = [189 x 255], matrix = [172 x 232], GRAPPA factor = 3, voxel size = 1.1 x 1.1 x 1.1 mm^3^, 126 slices, interslice distance = 0 mm, bandwidth = 1,540 Hz/px, echo spacing = 0.75 ms. Slice acquisitions were acquired for the whole brain, with interleaved slices, sagittal orientation, and anterior-to-posterior phase encoding. Opposite phase-encoded (i.e., posterior-to-anterior) slices with the same parameters were also acquired to perform distortion correction.

Whole-brain T1-weighted structural images were acquired as follows: TR = 2.53 seconds, TE = 1.65 ms, inversion time = 1.1 seconds, flip angle = 7°, 0.8 mm isotropic resolution, FOV = 240 x 240, GRAPPA factor = 2, and bandwidth = 1,200 Hz/Px. The participant’s physiological (i.e., heart rate using pulse oximetry and respiration using breathing bellows) signal recordings were collected throughout the scanning session.

Each neuroimaging session started each time the participant’s position was localized in the scanner and lasted until the next localization was conducted, usually after the participant left the scanner to rest, typically after 2 hours.

### 2.4 Neuroimaging preprocessing

Preprocessing steps were conducted at the level of MRI session and for all runs within that session, similar to our previous protocol, using AFNI (Yang et al., 2024). Details of the preprocessing steps are described in our previous study (Yang et al., 2024). In short, steps consisted of (1) de-spiking; (2) RETROspective Image CORrection (RETROICOR) (Glover et al., 2000) to regress out the effects of physiological (cardiac and respiratory) noise on data; (3) slice time correction; (4) distortion correction using opposite phase-encoded EPI; (5) motion correction; and (6) registering the anatomical dataset (T1) to a standard (MNI152_2009) template. Additional preprocessing steps included: (7) scrubbing any volume with motion >0.3 mm and had more than 5% outlier voxels; and (8) regressing out eroded cerebrospinal fluid (CSF) mask time course and motion parameters (3 translations, 3 rotations) per run, and band-pass filtering (0.01–0.1 Hz). Each fMRI run was then segmented into 1-minute segments for statistical analyses, similar to our previous work on advanced meditation (Yang et al., 2024).

### 2.5 Regions of interest (ROI) analysis

#### 2.5.1 Regional homogeneity analysis

Regional homogeneity (ReHo) is a measure of similarity in temporal activation pattern of a voxel and its nearby voxels (Zang et al., 2004). This measure of local functional connectivity within brain regions is a close derivative of underlying brain activity (Zang et al., 2004). A higher ReHo value indicates stronger local temporal synchrony among neighboring voxels, reflecting greater coherence in spontaneous neural activity patterns within a region. ReHo is interpreted as an index of local functional connectivity, should not be interpreted as an index of activation amplitude or task-evoked neural responses, and does not directly imply greater neural activation in the BOLD signal. In the context of EC, higher ReHo may reflect more coherent local spontaneous activity within a region, while lower ReHo may reflect reduced local synchrony and potentially more heterogeneous or attenuated regional dynamics. In this study, we defined a cluster size of 27 voxels and calculated the ReHo values for each fMRI segment. We standardized the ReHo values before smoothing the standardized ReHo maps using a 2 mm full-width half-maximum (FWHM) kernel.

We then used four different parcellation/segmentation schemes to define ROIs for subsequent ReHo analyses: (1) Schaefer-400 parcellation atlas for cortical areas (Schaefer et al., 2018); (2) 34-region Tian subcortex atlas for subcortical regions (Tian et al., 2020); (3) 54 Bianciardi brainstem atlas (Bianciardi et al., 2016); and (4) 10 Multi-Domain Task Battery (MDTB) functional cerebellar atlas (King et al., 2019). In total our parcellation/segmentation yielded 498 ROIs across the brain for each fMRI segment.

#### 2.5.2 Influence of neurotransmitter systems receptor density: Positron Emission Tomography (PET) neurotransmitter systems receptor density maps

We used a recently made available public neurotransmitter systems receptor density maps for 19 receptors and transporters, across 9 neurotransmitter systems by Hansen et al. (2022) at https://github.com/netneurolab/hansen_receptors. These include dopamine (D_1_, D_2_, DAT) noradrenaline (NAT), serotonin (5-HT_1A_, 5-HT_1B_, 5-HT_2A_, 5-HT_4_, 5-HT_6_, 5-HTT), acetylcholine (α_4_β_2_, M_1_, VAChT), glutamate (mGluR_5_, NMDA), GABA (GABA_A_), histamine (H_3_), cannabinoid (CB_1_), and opioid (MOR) receptors. Details regarding the neurotransmitter systems receptor density maps, each PET dataset, and their respective acquisition and limitations can be found elsewhere (Hansen et al., 2022). Volumetric PET images were registered to the MNI-ICBM 152 nonlinear 2009 (version c, asymmetric) template, averaged across participants within each study, and then parcellated and receptors/transporters with more than one mean image of the same tracer were combined using a weighted average (Hansen et al., 2022).

#### 2.5.3 Decoding of brain activity using data-driven Neurosynth cognitive maps

To provide a complementary approach for analyzing brain activity patterns with behavior in the absence of behavioral data, we examined the spatial similarity of ReHo maps with 123 meta-analytic brain maps derived from the Neurosynth database. Neurosynth generates probabilistic measurements that can be interpreted as a quantitative representation of the association between regional fluctuations in activity and psychological processes. Although Neurosynth’s database contains over 1,000 terms, we narrowed our focus to terms related to cognition and behavior, drawing from previous research (Hansen et al., 2022; Luppi, Hansen, et al., 2023; Yarkoni et al., 2011). Our selected set of 123 terms encompasses broad categories, ranging from attention and emotion, to more specific processes such as visual attention and episodic memory. The terms also include basic behaviors (e.g., eating and sleep) and emotional states (e.g., fear and anxiety). The full list of 123 cognitive terms used for decoding is provided in the Supplementary Excel file.

### 2.6 Statistical analysis

#### 2.6.1 ReHo brain activity

All statistical analyses were conducted using linear mixed-effects modeling to accommodate the hierarchical and temporally structured nature of the data, unless otherwise stated. Because each condition was segmented into consecutive 1-minute epochs to increase temporal resolution and statistical power, observations within the same run were not assumed to be independent, and inferential claims are framed as initial group-level estimates in a small sample. Adjacent segments may exhibit temporal autocorrelation due to slow fluctuations in neural and behavioral signals. Thus, we used a linear mixed-effects model with a first-order autoregressive AR(1) correlation structure to explicitly account for within-run temporal dependence (LMM-AR1) (Bolger & Laurenceau, 2013; Hamaker et al., 2005; McNeish & Hamaker, 2020). In this model, task condition was specified as the fixed effect of interest. Random intercepts were included for each run nested within participant to account for both between-participant variability and run-level differences within participants. The AR(1) structure models the expected decay in correlation across adjacent 1-minute segments within each run, allowing reasonable inference while retaining all segmented observations.

Given that our design comprised two control conditions and one experimental condition, we first examined differences between the two control conditions (counting vs memory). This initial comparison assessed whether the control conditions could be treated as statistically comparable with respect to the outcome measure. Given the absence of meaningful differences between control conditions, the two control conditions were subsequently pooled and contrasted against the EC condition. Pooling control conditions increased statistical power for detecting experimental effects while preserving the hierarchical and temporal structure of the data.

In addition to the group-level LMM-AR1 analysis, we also conducted supplementary participant-level LMM-AR1 analyses for each participant separately, using identical methodology given the substantial heterogeneity across participants in meditation experience (estimated 9,000 to 35,000+ lifetime hours), age (32–66 years), and phenomenological profile. These analyses allow assessment of whether group-level EC-related ReHo patterns reflect convergent signatures across individuals or are disproportionately driven by any single participant (Supplementary Fig. S1).

As additional robustness analyses, we also conducted supplementary pairwise comparisons between the experimental condition and each control condition separately (Supplementary Fig. S2). These analyses were performed to verify that the pooled-control effect was not driven disproportionately by a single control condition and to assess the consistency of the experimental effect across control contexts. We used Dunnett’s test to compare the difference between EC and control conditions, with EC as the reference group controlling for multiple comparisons using Bonferroni correction for k = 2 comparisons. For ease of interpretation, all coefficients were flipped to retrieve the contrast of EC against the control conditions.

As an additional complementary robustness check, we fit a conventional linear mixed-effects model that does not impose an explicit temporal correlation structure (LMM). In this supplemental model, random intercepts were specified for participants and for runs nested within participants, capturing the hierarchical structure of the data while implicitly assuming conditional independence between segmented observations within a run. Results from this analysis are reported in the supplementary results for completeness and transparency (Supplementary Fig. S3). The AR(1)-based model is considered the primary analysis because it more appropriately reflects the temporal dependence introduced by segmenting continuous runs into adjacent time windows. All LMM-AR1 and LMM analyses were conducted in *R* (R Core Team, 2022) loading on R Studio v2022.12.0.353.20 (Posit Team, 2022).

LMM-AR1 was used to analyze ReHo differences between EC and control conditions. Corrections for multiple comparisons across models were applied using false-discovery rate (FDR) across all 498 ROIs, with significance defined as *p_FDR_* < 0.05.

#### 2.6.2 Influence of neurotransmitter systems receptor density: Partial least squares correlation

We used partial least squares correlation (PLSC) analysis to explore the multivariate relationship between neurotransmitter systems receptor densities and EC delta-weighted-degree connectivity maps. PLSC is an unsupervised multivariate statistical technique that decomposes two datasets into orthogonal sets of latent variables with the goal of maximizing covariance between them (Krishnan et al., 2011). In this context, the latent variables consist of receptor weights, EC-control weighted-degree weights and a singular value that represents the covariance between receptor distributions and weighted-degree connectivity that is explained by the latent variable.

EC delta-weighted-degree maps were created by first thresholding all negative-weighted connectivity and self-connections to zero, followed by summing each region’s positive connection weights to all other regions. We only use cortical atlas for this analysis, similar to previous research on chemoarchitecture of the brain (Hansen et al., 2022; Luppi, Hansen, et al., 2023). Segment-level weighted-degree vectors were then averaged across all segments within each task, producing a single weighted-degree profile per participant per task. For each participant, the delta-weighted-degree map was calculated as the difference between EC and each control task, that is, EC-Counting, and EC-memory. Finally, participant-level delta-weighted-degree profiles were then averaged across the three participants to obtain the two delta-weighted-degree maps, EC-Counting, and EC-memory.

By projecting the original receptor density and weighted-degree data onto the corresponding latent variable weights, we computed receptor and EC-difference scores, assigning each brain region a specific score for both. Receptor loadings were then calculated as the Pearson correlation between neurotransmitter systems receptor densities and receptor scores, with a similar process used to compute EC-difference loadings (weighted-degree map and EC-difference scores). Importantly, PLSC does not (1) infer causal links between receptors and EC, (2) establish univariate associations between specific receptors and EC, or (3) rule out the possibility of additional connections between receptors and EC.

We assessed the significance of each latent variable’s singular value using permutation spin-testing (Alexander-Bloch et al., 2018; Markello & Misic, 2021; Vasa & Misic, 2022). For each map, parcel coordinates were projected onto the spherical surface and then randomly rotated and original parcels were reassigned the value of the closest rotated parcel (10,000 repetitions) (Vazquez-Rodriguez et al., 2019). In addition to preserving the distribution of cortical values, this null model also preserves the spatial autocorrelation present in the data.

#### 2.6.3 Contextualization of brain activity with Neurosynth cognitive maps

We used the *continuous.CorrelationDecoder* function from the NiMARE python package to contextualization of brain activity with Neurosynth cognitive maps (Salo et al., 2023). We first fetched Neurosynth dataset and converted it to NiMARE dataset. Following, we retrieved the 123 cognitive terms from the database. We also computed delta-ReHo maps (EC-Control, EC-counting, and EC-memory) for decoding. Finally, we used the *CorrelationDecoder* function to decode the association between the unthresholded delta-ReHo maps and cognitive maps.

## 3 Results

### 3.1 EC phenomenology

Demographics of participants (N = 3) are provided in Methods. Participant endorsement of EC phenomenology is presented in Figure 1. Here, we summarize the key phenomenological features associated with the setup, onset, EC, exit, and afterglow.

**Fig. 1.**
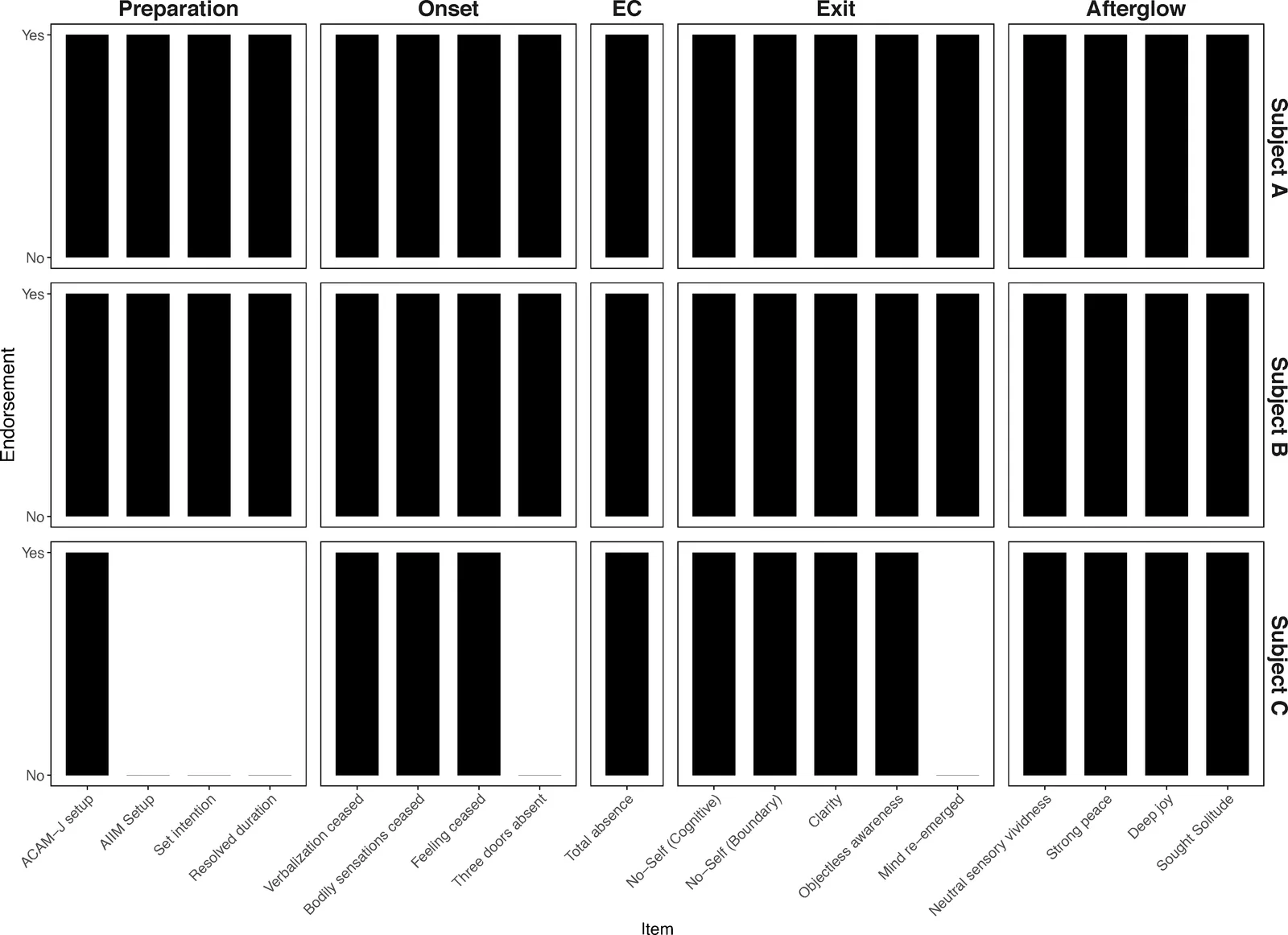
Endorsement of EC phenomenology for each participant. Participants all endorsed the phenomenological items of EC presented here, except Subject 4. Although previously trained to set up EC using ACAM-J, Subject 4 had since developed an alternative approach and no longer required ACAM-J for setup. As such, the phenomenology for various components throughout EC differ slightly for Subject 4. ACAM-J = Advanced concentration absorption meditation-jhana, AIIM = advanced investigative insight meditation.

***Setup.*** Participants typically used advanced absorption concentration meditation (ACAM) to prepare for EC. ACAM is a series of meditative absorption states accompanied by blissful sensations, emotional happiness, and equanimity, followed by an increasing reduction of input from the physical environment and the reduction of contents of consciousness to a bare minimum experience. One participant who had previously relied on ACAM for preparation reported no longer requiring ACAM to enter EC. Additionally, two of the three participants (Subjects A and B) incorporated elements of advanced investigative insight meditation (AIIM), that is, a neutral observation of phenomena, as part of their preparatory routine. Subject C, who had developed an alternative preparatory approach no longer requiring ACAM-J, did not incorporate AIIM in the same manner (see Fig. 1).

***Onset.*** Participants consistently reported that internal verbalization (thinking), body sensations, and feeling ceased during the onset of EC. Two of the three participants (Subjects A and B) reported no primary experiential emphasis on incongruence, the transient nature of experience, or self-lessness (features typically present during momentary cessations). Subject C, whose preparatory practice differed, reported a distinct onset profile consistent with his alternative approach (see Fig. 1).

***EC.*** During EC, participants reported the absence of reportable phenomenal experience.

***Exit.*** Upon emerging from EC, all participants reported that biographical memory and ego-centered cognitive processing were typically absent, and they did not perceive any separation between themselves and the environment. Participants consistently reported a vivid clarity of mind and an experience of objectless awareness. Two of the three participants (Subjects A and B) reported the return of mental activity in a specific sequence: first thought, then bodily sensation, and finally internal verbalization. Subject C reported a somewhat different re-emergence pattern consistent with his distinct preparatory and entry approach (see Fig. 1).

***Afterglow.*** After EC, participants consistently reported typically experiencing intense sensory vividness, accompanied by a strong and lasting peace. They all also reported typically being inclined to solitude, and many described a deep, abiding joy.

### 3.2 Brain activity during EC: Regional homogeneity (ReHo)

Complete results for significant ROIs, including test statistics, degrees of freedom, effect sizes, and pFDR, are reported in the Supplementary Excel file. Given the scale of the parcellation (498 ROIs), we present a narrative summary of the principal findings. Refer to the Supplementary Excel file for the full results.

Results are visualized in Figure 2a. Here, we summarize the principal findings. Linear mixed-effects models with an AR(1) autocorrelation structure (LMM-AR1) revealed that relative to the combined control conditions, EC was associated with higher ReHo values in the left visual cortex, posterior cortex, right dorsomedial prefrontal cortex (dmPFC), and the viscero-sensory-motor brainstem nuclei complex. Lower ReHo during EC was observed in cortical and subcortical regions, including the left insula, orbitofrontal cortex (OFC), ventral prefrontal cortex (vPFC), right somatomotor cortex, hippocampus, as well as brainstem nuclei, including the cuneiform nucleus, superior colliculus, and parabrachial pigmented nucleus complexes of the ventral tegmental area (VTA-PBP).

**Fig. 2.**
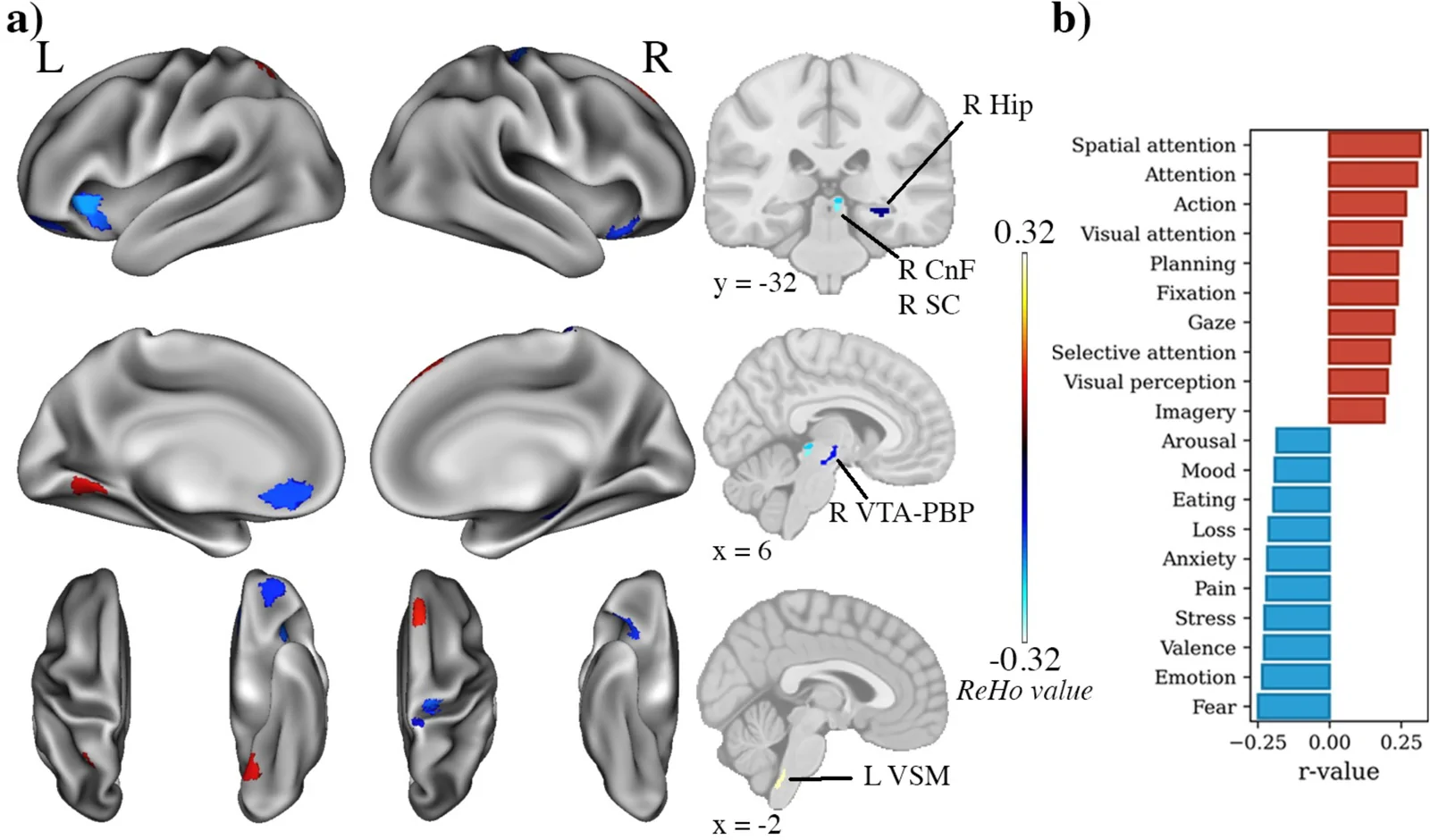
Differences in regional homogeneity values (ReHo) between EC and control conditions using a linear mixed-effects model with a first-order autoregressive correlation structure (LMM-AR1). (a) The figure shows the significant ReHo in the lateral, medial, dorsal, and ventral planes. The bottom-left pair shows the dorsal and ventral views of the left hemisphere, and the bottom-right pair shows the dorsal and ventral views of the right hemisphere, respectively. EC, compared to the counting control condition, demonstrated higher ReHo values in the left visual cortex, posterior cortex, right dorsomedial prefrontal cortex (dmPFC), and the viscero-sensory-motor brainstem nuclei complex (VSM), and lower ReHo values the left insula, orbitofrontal cortex, ventral prefrontal cortex, right somatomotor cortex, hippocampus, as well as brainstem nuclei, including the cuneiform nucleus (CnF), superior colliculus (SC), and parabrachial pigmented nucleus complexes of the ventral tegmental area (VTA-PBP). (b) Neurosynth decoding analysis revealed that these patterns of brain activity were related to cognitive processes associated with perception (e.g., attention, spatial attention, visual attention) and least likely with mental suffering and psychological distress (e.g., fear, stress, loss, pain).

Additionally, we also report subject-level contrasts (Supplementary Fig. S1), contrasts between EC and each control condition separately (Supplementary Fig. S2a, b), and contrasts using LMM without AR1 (Supplementary Fig. S3a–d).

### 3.3 Neurotransmitter systems receptor density: Partial least squares correlation (PLSC)

PLSC extracted one significant latent variable relating neurotransmitter systems receptor densities to delta-weighted-degree maps, explaining 98.85% of the covariance between the two datasets (p_spin_ = 0.023, one-tailed). Additionally, a positive correlation was found between the latent variables (r = 0.42, p < 0.001, Fig. 3a). We also computed the loadings for each receptor (Fig. 3b) and delta-weighted-degree map (Fig. 3c), where positively loaded receptors co-vary with positively loaded delta-weighted-degree in positively scored brain regions and similarly for negative loadings (Hansen et al., 2022). Projecting the neurotransmitter systems receptor density patterns back onto the delta-weighted-degree weights reflects how well a brain area exhibits the receptor and delta-weighted-degree weighted pattern, referred to here as ‘receptor scores’ and ‘delta-weighted-degree scores’, respectively (Fig. 3d, e).

**Fig. 3.**
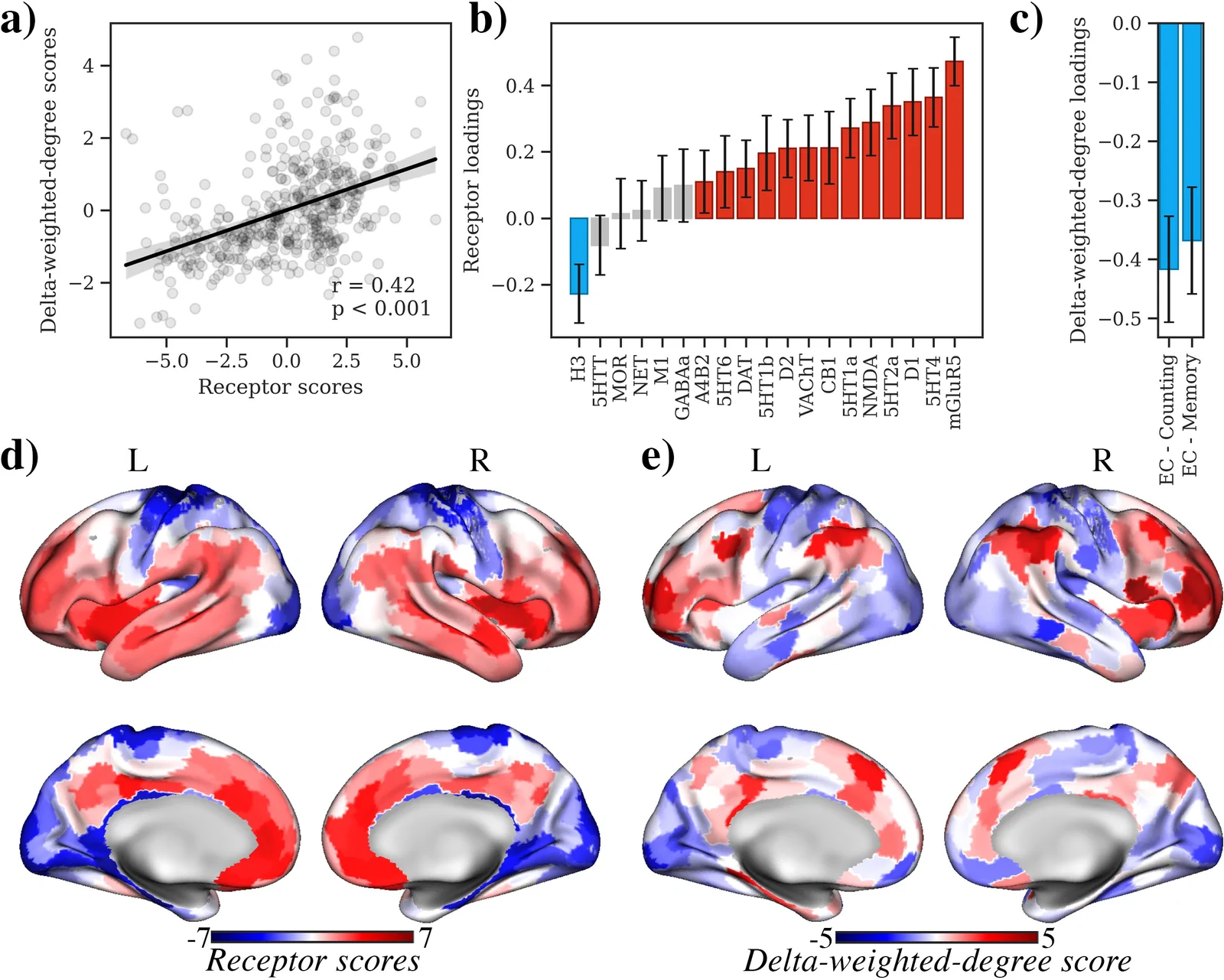
Spatial relationship between brain activity during EC and neurotransmitter receptor density. Only partial least squares (PLS) component 1 was significant. (a) Scatter plot shows the positive correlation between the latent receptor scores and latent delta-weighted-degree scores (r = 0.42, p < 0.001). (b) The bar chart displays the loadings of different neurotransmitter receptors on PLS component 1, with H_3_ receptors showing negative loadings while mGluR_5_, 5HT4, D1, 5HT_2A_, and NMDA showing positive loadings. (c) The bar chart shows the loadings of delta-weighted-degree maps between task conditions, with negative loadings on the difference between EC and counting (EC – counting) and memory (EC – memory). (d) Spatial distribution of coactivation between receptors (‘receptor scores’) and (e) delta-weighted-degree (‘delta-weighted-degree scores’). Red areas indicate regions with positive delta-weighted-degree scores, while blue areas represent regions with negative delta-weighted-degree scores.

Metabotropic glutamate receptor 5 (mGluR_5_), serotonin 5HT_4_, and dopamine D_1_ displayed the greatest positive loadings while histamine H_3_ displayed the only negative loading (Fig. 3b). Non-significant loadings include serotonin transporter 5HTT, mu-opioid receptor MOR, norepinephrine transporter NET, and GABA_A_. Both EC-Counting and EC-Memory delta weighted-degree maps have similar negative loadings (Fig. 3c). This suggests that the combination of H_3_ receptor distributions co-vary with EC-related weighted-degree values in unimodal regions (Fig. 3d). EC reduces connectivity where mGluR_5_/5HT_4_/D_1_ receptors are abundant and increases connectivity where H_3_ receptors are abundant, relative to both control tasks. Examining the brain scores, transmodal regions (PFC, PCC, temporal areas), where mGluR_5_/5HT_4_/D_1_/NMDA receptors are rich, decrease connectivity during EC relative to both controls (Fig. 3e). In contrast, primary sensory and motor cortices where H_3_ receptors are plenty, increase connectivity during EC. Collectively, these results demonstrate a direct link between whole-brain molecular receptor distributions and EC.

### 3.4 Neurosynth data-driven cognitive terms decoding analysis

***Across both counting and memory control conditions*****.** Correlation analysis revealed that EC was positively associated with action, attention, fixation, gaze, planning, spatial attention, visual attention, and visual perception. These terms are associated with perception and planning. In contrast, negative associations were found with terms such as anxiety, arousal, eating, emotion, fear, loss, mood, pain, stress, and valence. These terms are associated with mental suffering and psychological distress. Results are visualized in Figure 2.

***EC vs counting control condition*****.** EC was additionally positively associated with imagery and object recognition.

***EC vs memory control condition*****.** EC was additionally positively associated with selective attention and efficiency.

Full correlations for the 123 cognitive terms are reported in the Supplementary Excel file.

## 4 Discussion

Using intensively sampled within-individual design of ultra-high-resolution 7T functional MRI, we present an initial and first neural characterization of extended cessation (EC), a rare meditative state defined phenomenologically by the reported absence of perceptual and affective experience. We adopted an intensive within-subject, intensive-sampling design (N = 3), analogous to the rigorous small-N primate study designs and small-N human case studies to prioritize depth of characterization and internal validity over population-level generalization (Becker, 2023; Milne et al., 2018).

Prevailing neuroscientific frameworks propose that consciousness involves integrated brain activity across functionally segregated areas (Alkire et al., 2008), though the precise neural mechanisms and theoretical accounts of consciousness remain deeply debated (Mashour et al., 2020; Mudrik et al., 2025; Seth & Bayne, 2022; Tononi et al., 2016). The present findings are interpreted in the context of this theoretical plurality, and are not intended as a definitive test of any theory of consciousness. Because EC was examined using intensive, repeated measurements within individuals, we used LMM-AR1 to account for temporal autocorrelation of observations. Under this statistical framework, EC showed higher spontaneous brain activity within unimodal sensory regions, particularly visual cortex, posterior cortex, and viscerosensory-motor brainstem nuclei. In contrast, EC was associated with lower ReHo in several transmodal cortical regions, including insula, OFC, PFC, hippocampus, as well as in several brainstem nuclei (cuneiform nucleus, superior colliculus, and VTA-PBP). Said another way, EC may reflect a neural state in which perception is reorganized in the brain, while higher-order integrative, affective, and memory systems are attenuated. Such a configuration is consistent with a state of reduced representational and evaluative processing, while preserving basic sensory and physiological processes.

We interpret increased activity in visual and posterior cortical regions as unlikely to reflect processing of external sensory input. Rather, this pattern is more consistent with advanced meditative states in which perceptual representations are stabilized in the absence of valuation or self-referential processing (Chowdhury et al., 2025; Yang, Chowdhury, et al., 2025; Yang et al., 2024). The observation of elevated ReHo in sensory regions during EC warrants careful interpretation, as it is counterintuitive with the phenomenological definition of EC as involving complete cessation of sensory and phenomenal experience. We emphasize that ReHo reflects local temporal synchrony, not experiential content or perceptual awareness. Increased ReHo in visual and posterior cortical regions does not entail that visual experience is ongoing during EC. One possible account, consistent with active inference frameworks (Laukkonen et al., 2025; Parr et al., 2022; Tal et al., 2026), is that the attenuation of transmodal systems that ordinarily impose top-down precision-weighting on sensory representations allows intrinsic local synchrony in unimodal regions to persist or consolidate without generating phenomenal content. Under this account, the absence of phenomenal experience may arise not from suppression of sensory-region activity, but from the decoupling of sensory-region dynamics from the higher-order integrative systems required to transform brain activity into conscious percepts. We acknowledge, however, that this account remains speculative, and that reconciling elevated sensory-region synchrony with the claimed complete absence of phenomenal experience require future work combining multi-metric neuroimaging with more fine-grained phenomenological probing of the EC transition.

Greater activity in viscerosensory-motor nuclei suggest preservation of basic bodily regulatory and sensorimotor integrative functions necessary for physiological stability during EC. The viscerosensory-motor nuclei play a central role in coordinating autonomic regulation, interoceptive signaling, and basic sensorimotor integration, supporting core functions such as cardiovascular and respiratory control, as well as maintaining baseline arousal and homeostasis (Cauzzo et al., 2022; Edlow et al., 2024; Hansen et al., 2024; Mohebbati et al., 2021; Singh et al., 2022). Increased activity within these nuclei during EC likely reflects a stabilized physiological state in which fundamental bodily processes remain intact despite reduced engagement of higher-order cortical systems.

In contrast, reduced activity in the cuneiform nucleus, superior colliculus, and VTA-PBP during EC is consistent with attenuation of brainstem systems supporting action readiness, sensory orienting, and motivational salience (Edlow et al., 2024; Hansen et al., 2024). These structures are thought to support rapid engagement with behaviorally relevant stimuli, including shifts in attention, motor preparation, and dopaminergic signaling related to salience and incentive value. This interpretation is consistent with the broader pattern of reduced activity across the insula and extended limbic-prefrontal network, including the hippocampus, OFC, and PFC.

Within this broader pattern, reduced activity in OFC and PFC points to attenuation of higher-order systems involved in valuation, conceptual elaboration, self-referential processing, and goal-directed cognition. These regions form a core component of the brain’s transmodal system, supporting the construction of predictive, self-relevant models that link perception to goals, values, and future outcomes (Bernhardt et al., 2022; Bethlehem et al., 2020; Huang et al., 2023; Margulies et al., 2016). Their reduced engagement during EC suggests a weakening of top-down processes that normally impose structure, meaning, and behavioral relevance onto incoming sensory information. This interpretation is supported by the negative associations with affective and motivational Neurosynth terms that are consistent with diminished evaluative and goal-oriented processing. Together, these findings suggest that EC involves a downregulation of systems that ordinarily transform perception into self-relevant, value-driven, and action-oriented representations.

Reduced activity in the insula is especially notable because the insula plays a central role in integrating interoceptive signals with subjective feeling states and salience appraisal (Khalsa et al., 2018; Seeley et al., 2007; Uddin, 2015; Uddin et al., 2017). Rather than merely representing bodily sensations, the insula helps transform visceral and somatic inputs into felt, affectively toned experience that can be evaluated as pleasant, unpleasant, neutral, or self-relevant. Attenuation of insular activity during EC therefore suggests a reduction in the affective tagging of ongoing experience. In this view, sensory events may less strongly bound to feeling-tone, bodily urgency, and motivational significance. This interpretation aligns with the Neurosynth decoding results, which showed reduced associations with terms such as anxiety, arousal, emotion, fear, mood, pain, stress, and valence, collectively indicating diminished engagement of systems involved in psychological distress and affective evaluation.

The reduction in hippocampal activity further suggests that EC is associated not only with reduced affective evaluation, but also with diminished episodic contextualization and narrative continuity. The hippocampus contributes to situating ongoing experience within autobiographical and temporal context, linking present-moment contents to memory, expectation, and narrative selfhood (Addis et al., 2007; Borders et al., 2022; Eichenbaum, 2017). Reduced hippocampal involvement may suggest that experience during EC becomes less embedded within memory-based reconstruction and temporal self-continuity.

Together, these findings suggest that EC selectively disengages cortical-subcortical systems that ordinarily integrate perception with affective tone, mnemonic context, and self-relevant appraisal into a coherent, self-relevant experiential, or goal-directed cognition (Buckner & DiNicola, 2019; Buzsaki, 2026; Dohmatob et al., 2020; Lisman et al., 2017; Menon, 2023; Zhou et al., 2020). Importantly, this disengagement is accompanied by increased activity in visual and posterior cortical regions, as well as positive Neurosynth associations with attention, fixation, gaze, planning, spatial attention, and visual perception, and negative associations with fear, emotion, stress, pain, anxiety, and loss. This interpretation is further supported by exit phenomenology, in which objectless awareness precedes the gradual reconstitution of bodily sensation, thought, and finally internal verbalization. While such phenomenological reports cannot be directly mapped onto specific neural correlates, their consistency with our neural data provides a convergent descriptive context for interpreting EC-related brain dynamics.

To complement the group-level ReHo findings and address the possibility that averaging across participants may obscure individualized ReHo patterns given the small sample size, particularly given that Participant C employed a distinct entry approach and substantial heterogeneity across participants in meditation experience (estimated 9,000 to 35,000+ lifetime hours), age (32–66 years), we conducted participant-level LMM-AR1 ReHo analyses (Supplementary Fig. S1). These analyses revealed partial correspondence across the three participants, with increased ReHo in unimodal sensory and posterior cortical regions and decreased ReHo in transmodal and subcortical systems during EC, though with variation in spatial extent and effect magnitude across individuals. Yet, while the subcortical and transmodal regions showed substantial inter-individual variability, including reversal of direction in Subject C. This divergence raises the possibility that ReHo patterns may be sensitive to technique-specific differences in how EC is entered. Indeed, Participant C’s distinct entry approach relative to Participants A and B may have contributed to this divergence (see Methods). This participant-level perspective is consistent with recent demonstrations that neural signatures of subjective states can carry meaningful individualized structure (Lee et al., 2026), and highlights the value of intensive within-individual sampling designs for parsing common versus idiosyncratic features of rare meditative states. Disentangling technique-driven variability from individual trait differences will require larger samples spanning multiple entry techniques within the same individuals. Given the small sample, group-level findings should be treated with caution.

The neural profile observed during EC raises the possibility that some aspects of this pattern might be more parsimoniously explained by adjacent meditative states, such as deep non-evaluative absorption or non-dual awareness, neither of which necessarily involves complete phenomenal cessation. We consider this an important interpretive question, though one that currently lacks a direct empirical answer: to our knowledge, no published fMRI study has characterized the neural profile of deep non-evaluative absorption or non-dual awareness using comparable methodology, making a direct neural comparison impossible at this stage. The most informative empirical reference point available is our own prior work characterizing ACAM-J and AIIM using directly comparable 7T fMRI (Kumar et al., 2026; Vohryzek et al., 2025; Yang, Chowdhury, et al., 2025; Yang, Potash, et al., 2025; Yang et al., 2024) and EEG-based studies of momentary cessation events (Chowdhury et al., 2023; van Lutterveld et al., 2024, 2025). Lower ReHo in the hippocampus, insula, OFC, PFC, and brainstem motivational nuclei observed during EC is distinct from what was observed during deep concentrative absorption and advanced insight practice in those datasets, suggesting that the EC is not deep absorption with phenomenal consciousness. The post-EC phenomenology reported by participants further converges with complete cessation of phenomenal consciousness. That said, definitively distinguishing EC from adjacent states requires future within-subject comparative designs measuring EC alongside ACAM-J, AIIM, and non-dual awareness-type states within the same individuals.

EC can be situated relative to non-conscious states more commonly studied in neuroscience, such as anesthesia, sleep, and disorders of consciousness. While prior works on pharmacological interventions and pathological conditions often involve global suppression or structural disruption (Tanabe et al., 2020), EC appears to reflect a selective decoupling of systems that ordinarily integrate perception, affect, and self-relevance (Edlow et al., 2024; Redinbaugh & Saalmann, 2024; Seo et al., 2022; Wolff et al., 2021). Importantly, however, these other states were not measured within the same individuals, such comparisons should be understood as contextual and taken with caution. Nevertheless, our findings are consistent with the possibility that multiple neurobiological routes to phenomenological non-experience exist. Characterizing this configuration in a controlled, endogenous context allows the establishment of empirical boundary conditions for theories of consciousness and highlight the utility of advanced meditative states as natural experiments for dissociating wakefulness, brain organization, and phenomenal experience.

Contemporary theories of consciousness make distinct predictions about the neural conditions required for phenomenal experience. From a GNW perspective, conscious experience depends on the widespread integration and broadcasting of information across higher-order cortical systems (Mashour et al., 2020). The present ReHo findings are broadly compatible with this view insofar as EC involved reduced activity in regions associated with higher-order integration, including the PFC, OFC, insula, hippocampus, and thalamus. However, EC was not characterized by uniform cortical suppression. Rather, increased ReHo was observed in visual and posterior cortical regions, as well as in the right dmPFC and viscero-sensory-motor brainstem nuclei complex. This pattern suggests that the absence of phenomenal experience during EC cannot be explained simply by a global suppression of cortical activity, but instead may reflect selective disengagement of higher-order integrative systems.

IIT predicts that non-conscious states reflects reduced integration/differentiation or complexity across the brain (Huang et al., 2023; Luppi, Vohryzek, et al., 2023; Tononi et al., 2016). Based on our findings, EC does not appear to involve a uniform collapse of local neural organization. Instead, it shows a differentiated pattern in which some systems suppress activity while others enhance it. In particular, reduced activity in transmodal, affective, semantic, and action-related systems co-occurred with increased activity in visual, posterior, and brainstem regulatory regions. This suggests that absence of phenomenal experience during EC may arise not from reduced integration, but from a reorganization in which processing persists in selected subsystems while the regions that ordinarily integrate perception with feeling, memory, valuation, and action become attenuated.

Finally, according to predictive processing theory and AIF, loss of consciousness can arise from down-weighting the precision of sensory prediction errors (Parr et al., 2022) and a fundamental collapse of the brain’s generative model of reality (Laukkonen et al., 2025; Tal et al., 2026). Our findings are broadly compatible with an active inference account. The reduced activity in the transmodal DMN hubs, thalamus, and brainstem together with increased activity in unimodal systems is consistent with precision gating and “downward sampling cascade” where neural inference drops below the hierarchical threshold necessary to construct a conscious space. Together, these observations position EC as a theoretically informative boundary case, illustrating that loss of phenomenal experience can arise through selective network decoupling rather than global cortical suppression.

A unique contribution of this study is the link between EC and neurotransmitter receptor topography. Brain activity during EC co-varies with H_3_ receptor topography where regions showing increased ReHo during EC tend to be rich in H_3_ receptors, while regions showing decreased ReHo tend to be rich in mGluR_5_, 5HT_4_, and D_1_ receptors. H_3_ receptors regulate histamine release and exert inhibitory control over acetylcholine, dopamine, and norepinephrine, shaping arousal, attention, and sensory filtering (Esbenshade et al., 2008; Lin et al., 2011; Yu et al., 2018). EC’s coupling to H_3_-dense unimodal cortices may reflect a neurochemical substrate for the reduced modulatory drive from subcortical arousal systems during EC, consistent with the observed reduced ReHo in the VTA-PBP and cuneiform nucleus.

In contrast, the transmodal regions where ReHo is reduced during EC are rich in mGluR_5_/NMDA/D_1_ may allow habitual cognitive constraints to relax, enabling underlying intrinsic brain dynamics to emerge (Tal et al., 2026) and modulate the type of conscious experience (Zilles et al., 2015). Reduced ReHo in these receptor-rich regions aligns with the Neurosynth decoding results showing reduced associations with anxiety, emotion, stress, and self-referential terms during EC, and is consistent with an active inference account in which precision-weighting of higher-order generative models is reduced during EC.

Although these associations are correlational, they provide a plausible neurochemical context for the reproducible phenomenological aftereffects reported following EC and align with prior observations of reduced transmodal network activity observed during ACAM (Yang et al., 2024) and AIIM (Yang, Chowdhury, et al., 2025). However, several important caveats apply. The receptor density maps used here are derived from independent, normative PET cohorts, not from the present participants. Recent methodological work has highlighted that cross-cohort spatial correlations are vulnerable to inflated Type I error rates due to inherent spatial autocorrelation, necessitating caution when interpreting uncorrected spatial overlaps (Markello & Misic, 2021). Recent work highlights significant inter-individual variability in neurotransmitter receptor density that is inherently lost in group-averaged atlases (Hansen et al., 2026). Therefore, the finding that EC activity patterns co-vary with H_3_ receptor topography should be treated as exploratory and interpreted as hypothesis-generating. Definitive conclusions on the neurochemical mechanisms of EC will require studies that acquire both EC and PET receptor imaging within the same individuals.

Taken together, the present findings suggest that EC constitutes a qualitatively distinct meditative state characterized by the temporary absence of reportable phenomenal experience followed by a distinctive set of profound subjective aftereffects, including clarity, equanimity, and reduced mental suffering. Data-driven meta-analytic decoding using Neurosynth situates the EC brain state away from cognitive–affective domains associated with distress and self-referential processing and closer to perceptual and attentional domains. Importantly, these mappings do not imply benefit or mechanism but indicate that the neural configuration observed during EC dissociates from cognitive-affective signatures of psychological suffering (Sparby & Sacchet, 2025).

This study has several strengths, including whole-brain coverage at ultra-high spatial resolution and the use of conservative statistical models accounting for temporal autocorrelation. Nevertheless, there are several important limitations to consider. As the first empirical investigation of EC, the present findings should be regarded as preliminary and require replication. The small sample size reflects the rarity of the phenomenon but limits generalizability. While we compared EC to non-meditative states, a natural extension would be to compare EC to other meditative states to elucidate similarities and differences between EC and advanced forms of mediation. The absence of consciousness and self-reports during EC poses a challenge in understanding the subjective non-experience and its correlation with the neuroimaging findings. However, Neurosynth decoding offered robust evidence linking EC brain state to enhanced perceptual clarity, sensitivity, and the absence of mental suffering. These findings strongly suggest that significant brain reorganization occurs during EC to a degree that supports these subjective subsequent phenomena. The present study relies on ReHo as the primary neuroimaging metric. ReHo does not provide information about large-scale network dynamics, amplitude of neural fluctuations, or graph-theoretic properties of brain organization. Thus, future EC studies would benefit from evidence across complementary metrics including amplitude of low-frequency fluctuations, functional connectivity, or graph-theoretic measures.

We also acknowledge a potential concern regarding the use of button press to mark the entry and exit of EC. Performing a volitional motor response prior to entry and after re-emergence of EC mitigates but does not eliminate this concern as the button press may interfere or interact with the state. Based on post-session phenomenological reports, participants describe being able to enter EC within seconds of the button press so it does not interfere with the state. Moreover, we have removed 1 minute of volumes after entry and before exit to mitigate bleeding effects into EC. Nonetheless, the timing between the subjective experience of EC exit and the motor response introduces some uncertainty, and we cannot rule out that the motor preparation associated with button press represents a confound at the onset or re-emergence. Future studies may benefit from passive exit detection paradigms, for example, real-time monitoring of physiological or neural markers, to supplement or replace button press marking.

In conclusion, our results provide the first evidence for the neural correlates of EC. EC is marked by higher activity in unimodal regions with downregulation of transmodal and subcortical regions. These findings not only deepen our understanding of extended cessation but also provide a theoretically informative case for dissociating wakefulness, neural organization, and phenomenal experience. More broadly, EC reveals a selective “reset” capacity of the mind that attenuates self-referential burden while sharpening perceptual engagement, opening tractable neuromodulatory and behavioral routes to reduce psychological suffering and advance human flourishing.

## Supplementary Material

Supplementary Material 1

Supplementary Material 2

Supplementary Material 3

## Data Availability

Data may be requested from the corresponding author and are subject to the Massachusetts General Hospital Institutional Review Board’s guidelines and approval. All code used in this study may be made available by request from the corresponding author.
