# Supplementary material for: Endogenous suspension of phenomenal consciousness during extended cessation: Regional homogeneity analysis of multi-participant 7T fMRI of an advanced meditative endpoint
Source: Imaging Neurosci (Camb). 2026 Nov 5;4:IMAG.a.1443. doi: 10.1162/IMAG.a.1443 (PMC13742257; doi:10.1162/IMAG.a.1443)
Supplement: Supplementary file 1 [file IMAG.a.1443_supp1.pdf]

This dictionary file provides information regarding the contents within each Excel tab, summarizing significant findings presented in the paper.

ReHo-AR-LMM and ReHo-LMM: Contains significant Regional Homogeneity (ReHo) analysis results related to extended cessation across counting and memory tasks. The ReHo-AR-LMM assumes AR(1) autocorrelations in the model while ReHo-LMM does not assume any autocorrelation in the model. Included are Linear Mixed Model (LMM) statistics such as chi-square values, parameter estimates, standard errors, t-values, adjusted p-values, and FDR-adjusted chi-square p-values across 498 Regios of Interest (ROIs).

- **Control Condition:** Experimental condition used as the control (i.e., EC – memory and EC - counting).
- **Atlas:** Brain atlas used to define the ROI (Schaefer, Tian, Bianciardi, MDTB).
- **ROI:** Specific Region of Interest under analysis.
- **Network:** Functional brain network assignment of the ROI (e.g., DMN, FPN).
- **chisq:** Chi-square test statistic from the linear mixed model.
- **chisq.pval:** Raw p-value for the chi-square test.
- **Estimate:** Estimated effect size from the model.
- **SE:** Standard Error of the estimate.
- **TVAL:** T-statistic value from the model.
- **PVAL_ADJUSTED:** Bonferroni-adjusted p-value for t-test comparison (for EC vs memory/counting).
- **CHISQ_PVAL_ADJUSTED:** FDR-adjusted p-value for chi-square test (corrected across 498 ROI tests).

Neurosynth Tab: Features comprehensive correlations between ReHo values and 123 cognitive neuroscience terms from the Neurosynth database.

- **Feature:** Cognitive term from the Neurosynth meta-analytic database (e.g., “attention”, “memory”).
- **EC-Counting r / EC-Memory r:** Pearson’s correlation (r) between ReHo and Neurosynth feature map during for EC-counting and EC-memory contrasts.

PLSC Scores Tab: Presents delta-degree connectivity scores and receptor scores derived from Partial Least Squares Correlation (PLSC) analyses.

- **ROI:** Brain region.
- **Network:** Functional network label.
- **Hemisphere:** LH (left), RH (right).
- **LV1-X-scores / LV2-X-scores:** Delta-degree-connectivity scores.
- **LV1-Y-scores / LV2-Y-scores:** receptor profile scores.
