# Supplementary material for: Endogenous suspension of phenomenal consciousness during extended cessation: Regional homogeneity analysis of multi-participant 7T fMRI of an advanced meditative endpoint
Source: Imaging Neurosci (Camb). 2026 Nov 5;4:IMAG.a.1443. doi: 10.1162/IMAG.a.1443 (PMC13742257; doi:10.1162/IMAG.a.1443)
Supplement: Supplementary file 2 [file IMAG.a.1443_supp2.pdf]

Supplementary Information for:

Title: Endogenous suspension of phenomenal consciousness during extended cessation: Regional homogeneity analysis of multi-participant 7T fMRI of an advanced meditative endpoint

Authors

Winson F.Z. Yang[1,2], [+]Akila Kadambi[3,4], Ruby Potash[1,2], Terje Sparby[5,6,7], Matthew D. Sacchet[1,2]* (meditationadministration@mgh.harvard.edu)

[+]Denotes co-second-authors

Affiliations

[1]Meditation Research Program, Department of Psychiatry, Massachusetts General Hospital, Harvard Medical School, Boston, USA

[2]Athinoula A. Martinos Center for Biomedical Imaging, Department of Radiology, Massachusetts General Hospital, Harvard Medical School, USA

[3]Dept. of Psychiatry and Biobehavioral Sciences, David Geffen School of Medicine, University of California, Los Angeles, USA

[4]Brain and Creativity Institute, Dornsife College of Letters, Arts, and Sciences, University of Southern California, Los Angeles, USA

[5]Steiner University College, 0260 Oslo, Norway

[6]Department of Psychology and Psychotherapy, Witten/Herdecke University, 58455 Witten, Germany

[7]Integrated Curriculum for Anthroposophic Psychology, Witten/Herdecke University, 58455 Witten, Germany

Supplementary figures

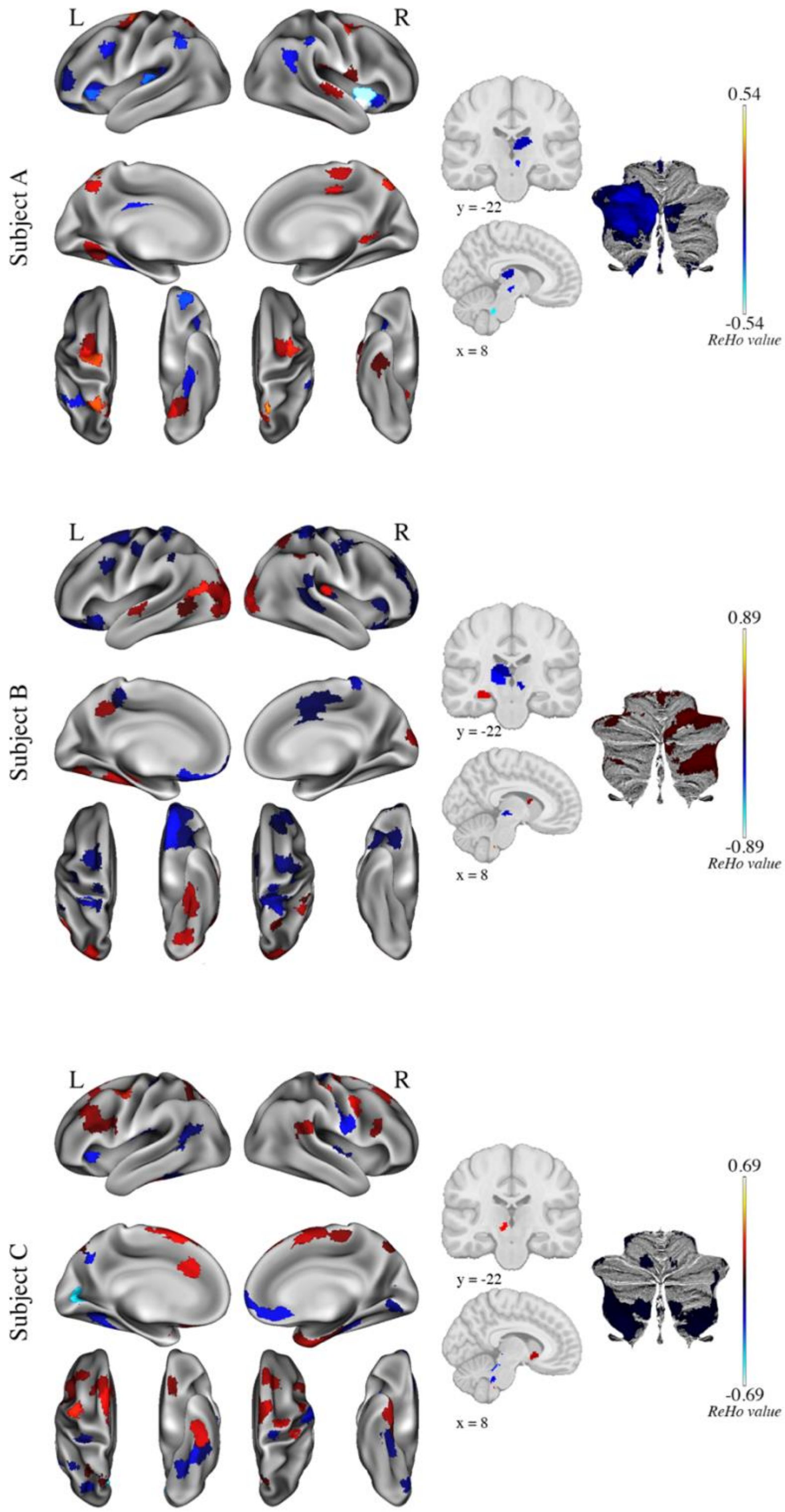

Figure S1. Subject-level differences in regional homogeneity values (ReHo) between extended cessation (EC) and control conditions using a linear mixed-effects model with a

first-order autoregressive correlation structure (LMM-AR1). The figure shows the significant ReHo in the lateral, medial, dorsal, and ventral planes. The bottom-left pair shows the dorsal and ventral views of the left hemisphere, and the bottom-right pair shows the dorsal and ventral views of the right hemisphere, respectively. At the individual level, Subject A generally showed higher ReHo in the visual and somatomotor cortex and lower ReHo values in the prefrontal, subcortical regions, and in the cerebellum. Subject B showed higher ReHo values in the visual regions, hippocampus, amygdala, and cerebellum, and lower ReHo values in the somatomotor, orbitofrontal cortex, and subcortical regions. Finally, Subject C demonstrated higher ReHo in the dorsomedial regions, temporal pole, nucleus accumbens, amygdala, and multiple brainstem nuclei although several right brainstem regions showed decreases, and decreased ReHo in the visual, somatomotor, posterior, orbitofrontal cortex, insula, and cerebellum.

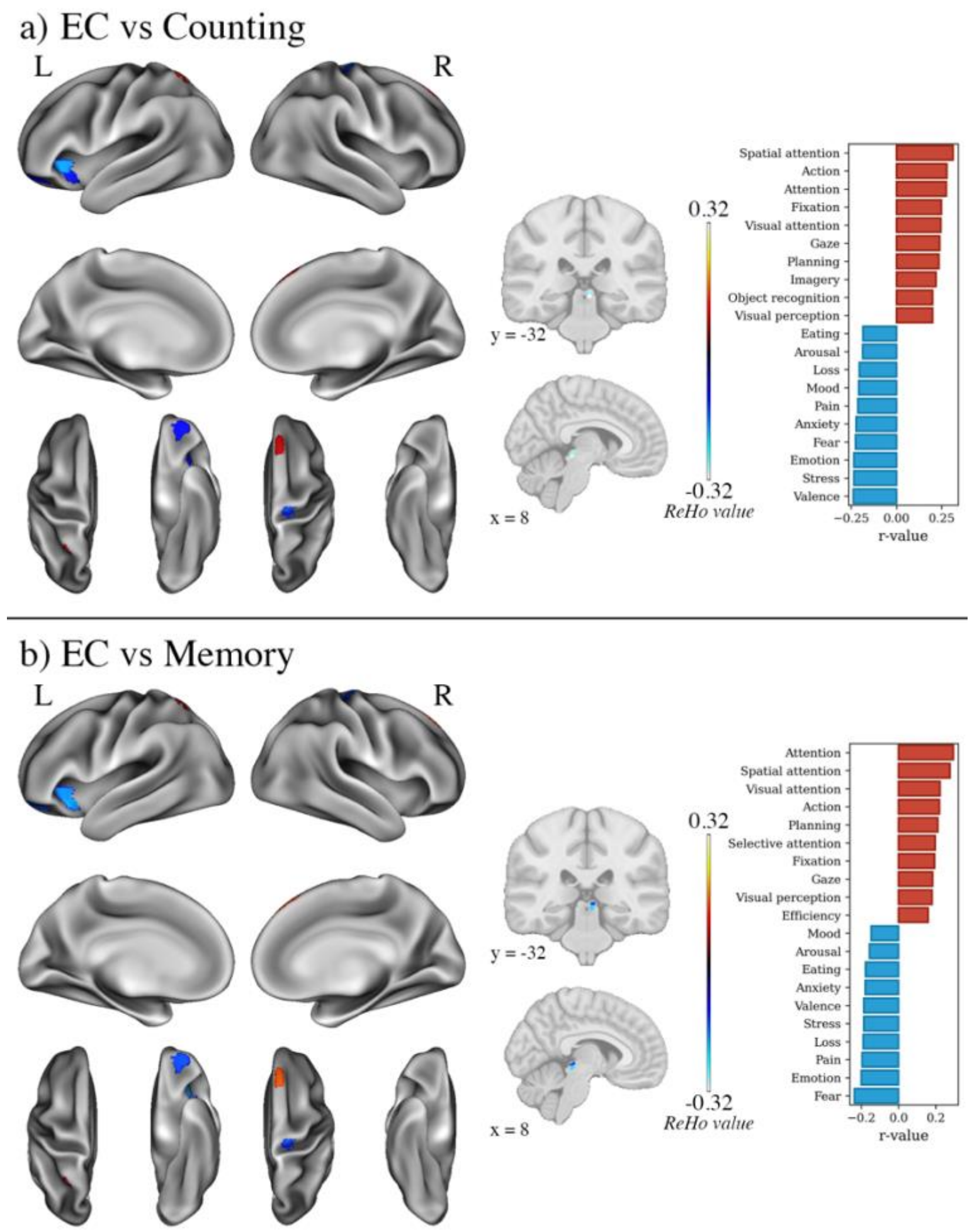

Figure S2. Differences in regional homogeneity values (ReHo) between extended cessation (EC) and control conditions using a linear mixed-effects model with a first-order autoregressive correlation structure (LMM-AR1). LMM-AR1 showed that (a) EC, compared to the counting control condition, demonstrated increased ReHo values in the right dorsomedial prefrontal cortex (dmPFC), and decreased ReHo in the left posterior cortex, insula, orbitofrontal cortex (OFC), prefrontal cortex (PFC), right somatomotor cortex, cuneiform nucleus, and superior colliculus. (b) Similarly, compared to the memory condition, EC displayed increased and decreased ReHo values in the same regions. Neurosynth decoding analysis revealed that these patterns of brain activity were related to cognitive processes associated with perception (e.g., attention, spatial attention, visual attention) and least likely with mental suffering and psychological distress (e.g., fear, pain, loss, stress, anxiety, mood).

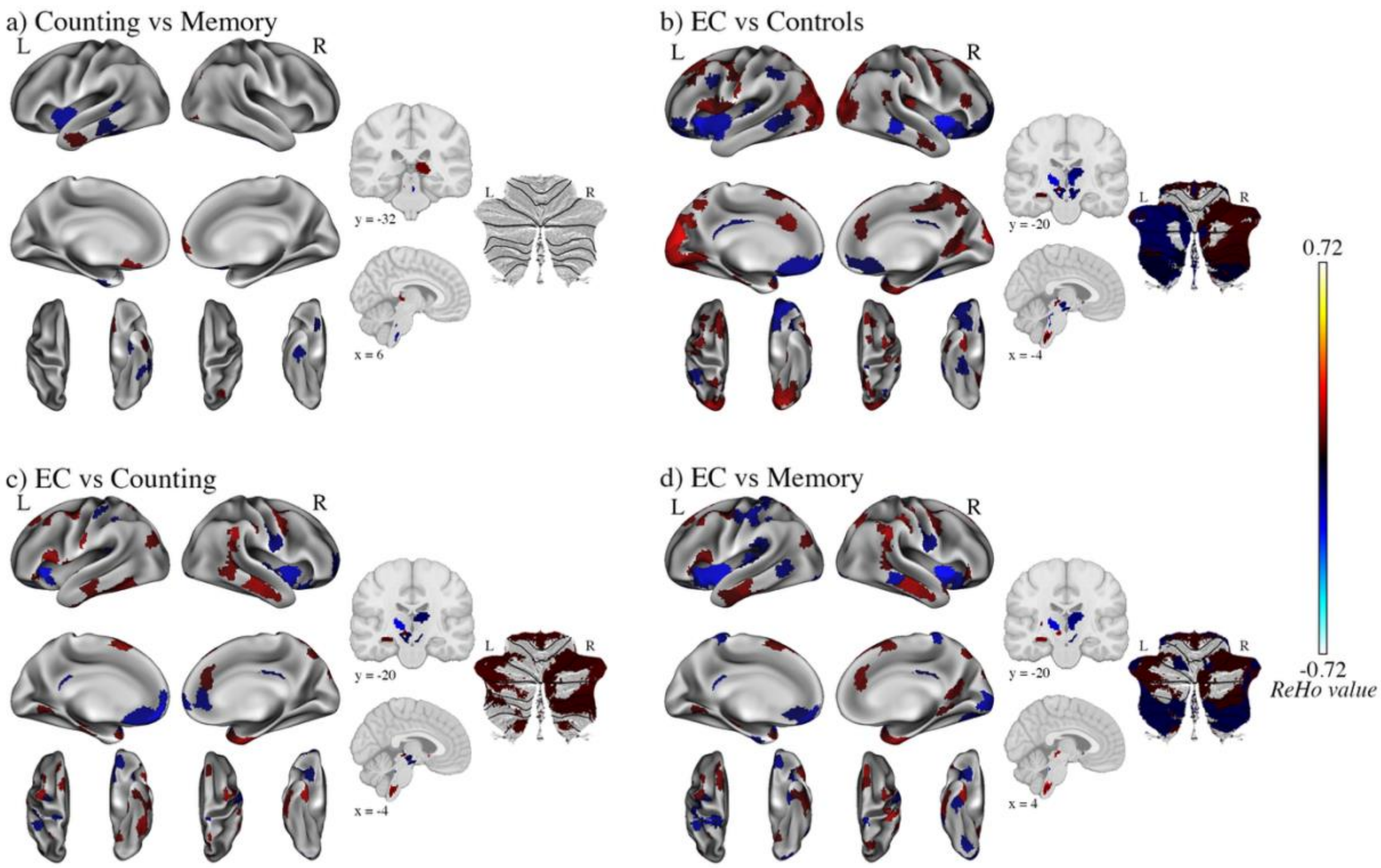

Figure S3. Differences in regional homogeneity values (ReHo) between EC and control conditions (LMM model without AR1). (a) Significant differences between the counting and memory control conditions are minimal, but constitutes cortical, subcortical, and brainstem regions. **(b)** Differences between EC and combined control conditions were mostly found in the visual and prefrontal cortices, subcortical limbic regions, brainstem nuclei, and cerebellum. **(c)** Differences between EC and **(c)** counting and **(d)** memory condition were mostly found in the prefrontal, temporal, insular, parietal, and somatomotor cortices, subcortical thalamic and limbic regions, brainstem nuclei, and cerebellum.
